# Comparison of ROX index (SpO_2_/FIO_2_ ratio/respiratory rate) with a modified dynamic index incorporating PaO_2_/FIO_2_ ratio and heart rate to predict high flow nasal cannula outcomes among patients with acute respiratory failure: a single centre retrospective study

**DOI:** 10.1186/s12890-022-02121-9

**Published:** 2022-09-16

**Authors:** Amit Kansal, Wei Jun Dan Ong, Shekhar Dhanvijay, Arbe Tisha Pepito Siosana, Loraine Mae Padillo, Chee Keat Tan, Monika Gulati Kansal, Faheem Ahmed Khan

**Affiliations:** 1 Department of Intensive Care Medicine, Ng Teng Fong General Hospital, Jurong Health Campus, National University Health System, 1 Jurong East Street 21, Singapore, 609606 Singapore; 2 Department of Respiratory Therapy, Ng Teng Fong General Hospital, National University Health System, 1 Jurong East Street 21, Singapore, 609606 Singapore

**Keywords:** Acute respiratory failure, HFNC, HFNC failure, Adult ICU

## Abstract

**Background:**

High flow nasal cannula (HFNC) is increasingly being used to support patients with acute respiratory failure (ARF) and to avoid need for intubation. However, almost one third of the patients do not respond and require escalation of respiratory support. Previously, ROX index (SpO_2_/FIO_2_ [SF] ratio/respiratory rate) has been validated among pneumonia patients to facilitate early recognition of patients likely to fail HFNC and therefore, benefit from timely interventions. However, it has been postulated that incorporation of PaO_2_/FIO_2_ (PF) ratio from arterial blood gas (ABG) analysis may better predict the outcome of HFNC compared to indices that utilizes SF ratio. Similarly, heart rate increase after HFNC therapy initiation has been found to be associated with HFNC failure. Therefore, we aimed to compare ROX index with a new modified index to predict HFNC outcomes among ARF patients.

**Materials and methods:**

This single centre 2-year retrospective study included ARF patients of varying etiologies treated with HFNC. The modified index incorporated heart rate and substituted PF ratio for SF ratio in addition to respiratory rate. We named the index POX-HR and calculated Delta POX-HR index as the difference pre- and post-HFNC initiation POX-HR. We also recorded ROX index at the time when post-HFNC initiation ABG was done (‘post-HFNC initiation ROX’) and calculated Delta ROX. HFNC success was defined as no need of escalation of respiratory support or discharged to ward within 48 h of HFNC initiation, or successful wean off HFNC for at least 12 h. Evaluation was performed using area under the receiver operating characteristic curve (AUROC) and cut-offs assessed for prediction of HFNC outcomes.

**Results:**

One hundred eleven patients were initiated on HFNC for ARF, of whom 72 patients (64.9%) had HFNC success. Patients with HFNC failure had significantly lower values for all the indices. At median of 3.33 h (IQR 1.48–7.24 h), Delta POX-HR demonstrated the best prediction accuracy (AUROC 0.813, 95% CI 0.726–0.900). A Delta POX-HR > 0.1 was significantly associated with a lower risk of HFNC failure.

**Conclusions:**

Our proposed modified dynamic index (Delta POX-HR) may facilitate early and accurate prediction of HFNC outcomes compared to ROX index among ARF patients of varied etiologies.

## Introduction

High flow nasal cannula (HFNC) is increasingly being used to support patients with acute respiratory failure (ARF) and to avoid need for intubation [1]. However, a significant number of patients do not respond and require escalation of respiratory support (28–38%) [2–4]. Since delayed intubation has been associated with poorer outcomes, early recognition of patients likely to fail HFNC can be immensely helpful in clinical decision making [5–7].

Previously, ROX index (SpO_2_/FIO_2_ [SF] ratio /respiratory rate) has been developed and validated to identify patients at low risk of HFNC failure among patients with hypoxemic respiratory failure due to pneumonia [2, 3]. The ROX index has been considered useful clinically because it requires few data points and is simple to calculate at the bedside. However, ROX index is not validated among patients with ARF other than pneumonia; the cut off for ROX values that can predict HFNC outcomes are shown to be variable among different patient populations [2, 3, 8–11].

Additionally, ROX index does not incorporate other commonly available clinical variables like heart rate (HR) and PaO_2_/FIO_2_ (PF) ratio. Increased HR is likely to be a surrogate for the sympathetic drive due to increased work of breathing. Normal compensated cardiovascular adaptation to acute hypoxemia involves increased cardiac output, mediated predominately by tachycardia, with only moderate augmentation of blood pressure [12]. The heart rate response to hypoxemia parallels the ventilatory response [13]. Tachycardia may also represent poor cardiac reserves. Tachycardia after HFNC therapy initiation has been found to be associated with HFNC failure[7, 14]. Goh et al. recently demonstrated that a modified ROX index (termed as ROX-HR) incorporating HR may be a better predictor of HFNC outcomes in ARF as well as preventive use in post-extubation setting when compared to ROX index alone [9].

It has been postulated that incorporation of PF ratio from arterial blood gas (ABG) analysis may better predict the outcome of HFNC compared to ROX index that utilizes SF ratio [15]. Studies have shown that the relationship of SF ratio and the PF ratio is not linear and may not fare well at higher FIO_2_ [16, 17]. Similarly, the fall of SpO_2_ and PaO_2_ is also not linear [18]. One previous study among pneumonia patients showed that PF ratio within 6 h of HFNC initiation was lower in HFNC failure group when compared to HFNC success group [7]. Many patients receiving HFNC for the respiratory failure are likely to have serial ABG analysis available before and after HFNC to evaluate the response and it may be prudent to utilize such additional available information [19]. However, no studies have evaluated modified ROX criteria by substituting PF ratio for SF ratio.

Previous studies have also highlighted that a change in indices over time may be a better predictor of HFNC outcomes than a value at any given time point [3, 9].

We planned this retrospective study to evaluate a modified dynamic index incorporating HR and substitution of PF ratio for SF ratio in addition to respiratory rate pre- and post-HFNC initiation in comparison with ROX index to predict outcomes of HFNC therapy in patients with ARF of varied etiologies. We also recorded ROX index at the time when post-HFNC initiation ABG was done (‘post-HFNC initiation ROX’) and calculated Delta ROX for further comparison.

## Methods

### Study design and patient population

The study was conducted in a mixed adult intensive care unit (ICU) of a tertiary care public hospital in Singapore. We included all adult patients admitted to the ICU who were initiated on HFNC over a two-year period from January 1, 2018 to December 31, 2019. For subjects with multiple ICU admissions and HFNC usage within one hospitalization, only the first HFNC episode was included. Data were collected retrospectively from the electronic medical records. The National Healthcare Group (NHG) Domain-Specific Review Board (DSRB) approved the study with a waiver of informed consent due to the non-interventional retrospective study design (NHG DSRB reference number—2020/01167).

Patients were included if they were older than 18 years and were initiated on HFNC for ARF. Patients with concomitant hypercapnia (PaCO_2_ more than 45 mmHg) in the pre-HFNC ABG analysis were also included. We excluded patients with a ‘do not resuscitate or do not intubate’ order. Patients were excluded if there was an urgent need to intubate within 2 h after HFNC initiation, since we considered that such patients were likely to be too sick to be trialled on HFNC. We didn’t include the patients who were cycled between NIV and HFNC. This subgroup of patients is being studied as a separate sub-group in various previous studies in view of distinct risks and benefits of NIV cycling compared to HFNC alone [4, 20]. We also excluded the patients on beta-blocker and beta-agonist therapy due to interference with heart rate response.

### Clinical management and definition of HFNC failure

Airvo 2™ (Fisher & Paykel Healthcare, Auckland, New Zealand) was used for providing HFNC therapy. HFNC was initiated at a minimum flow of 50 L/min (50–60 L/min), titrating FIO_2_ to achieve an oxygen saturation of ≥ 92% as per routine practice in our unit. Usually, HFNC would be reduced to 30–40 L/min once FIO_2_ is stable at 30–40% and thereafter switch to standard oxygen therapy in next 6–24 h if clinically stable. Use of HFNC as well as the need for escalation of respiratory support to intubation or non-invasive ventilation (NIV) was decided by the treating clinicians based on their clinical judgement as deemed appropriate. The study ICU is always covered by trained intensive care consultants and respiratory therapists. We defined HFNC success as no need for escalation of respiratory support to intubation or NIV, or discharged to ward (off HFNC) within 48 h of HFNC initiation, or successful wean off HFNC for at least 12 h. HFNC failure was defined as need to escalate to intubation or NIV within 48 h of HFNC application for respiratory causes only (e.g., patients requiring intubation for surgical intervention in operating theatre were NOT counted as HFNC failure). As stated earlier, we excluded patients who were cycled between NIV and HFNC. We used 48 h as an endpoint acknowledging that an intubation later than 48 h may not be related to the early physiological parameters. Patients were followed up till death or hospital discharge.

### Data collection

We collected data for patients’ demographic characteristics, admission diagnosis (medical/ surgical), Sequential Organ Failure Assessment (SOFA) score, Acute Physiology and Chronic Health Evaluation II (APACHE II) score at the time of ICU admission, Glasgow coma score (GCS), co-morbidities, body mass index (BMI), treatment limitation (before initiation of HFNC therapy).

The following clinical and ABG parameters were collected for the time period prior to and after HFNC initiation: respiratory rate (RR), HR, pH, bicarbonate (mmol/L), partial pressure of carbon dioxide (PaCO_2_, in mmHg), partial pressure of oxygen (PaO_2_, in mmHg), PF ratio, SpO_2_ (%), and SF ratio. In our institution, an ABG analysis was usually conducted during HFNC therapy in most cases prior to HFNC initiation and during HFNC to assess oxygenation and other parameters. Finally, outcome data of mortality (ICU and hospital) were collected.

We recorded ROX index just prior to HFNC initiation and at 2, 6, 12, 18 and 24 h afterwards. ROX index has been defined as SF ratio/ respiratory rate.

### Modified index description

The modified index predicting HFNC outcomes was calculated by incorporating HR and substitution of PF ratio for SF ratio in addition to RR. HR was placed in the denominator as it has an inverse association with HFNC success [7, 9]. We named the index POX-HR, defined as PF ratio/ [RR*HR] and multiplied by a factor of 100. We recorded the RR and HR at the timepoint of pre- and post- HFNC ABG analysis and calculated the respective POX-HR indices and recorded ‘Delta POX-HR’ index as the difference between two values. We also recorded ROX index at the time when post-HFNC initiation blood gas analysis was done (‘post-HFNC initiation ROX’) and calculated Delta ROX (difference between ROX index just prior to initiation of HFNC versus post-HFNC initiation ROX).

### Statistical analysis

Categorical variables were reported as frequencies and proportions and were compared using the Chi-square test. Non-parametric data was reported as median (interquartile range, IQR) and compared using the median test. Cut-offs for the various indices, rounded off to the nearest 0.01, were chosen to maximise the sum of sensitivity and specificity based on the receiver operating characteristic (ROC) curves (to satisfy the highest value for Youden index). From these cut-offs, Kaplan–Meier (KM) plots for HFNC success were determined and compared using the log-rank test. Univariate and multivariate Cox proportional regression analysis was performed to evaluate POX-HR indices. Additionally, we identified the cut off thresholds for HFNC failure based on 90% specificity. Statistical difference was considered significant at *p* ≤ 0.05. All statistical analyses were performed using the SPSS software (version 23.0 SPSS Inc., Chicago, Illinois, USA).

## Results

A total of 200 patients received HFNC during the study period. 89 of these patients were excluded: fifteen patients did not have pre or post HFNC initiation ABG, 36 patients had HFNC support for less than two hours including three patients who were intolerant, 16 were cycling with NIV therapy and 21 patients had a ‘do not resuscitate or intubate’ order. One patient was on beta-blocker therapy, none of the patients required beta-2 agonists.

Remaining 111 patients were initiated on HFNC for ARF and were included in the study analysis (Tables 1, 2). Pneumonia was the most common primary diagnosis (75.8%). The median FIO_2_ requirement at time of HFNC initiation was 40.0% (33.0–80.0%) with a median PF ratio of 162.5 (IQR 112.0–228.0). Fifty-one patients (45.9% patients) required an FIO_2_ of more than 40%. The RR was 26 per minute (21–30/min) before HFNC initiation. These patients had an APACHE II score of 20 (IQR 15–27) and SOFA score of 6 (IQR 4–9) at ICU admission. The median time of ABG taken before and after the initiation of HFNC was 2.38 h (IQR 1.51–5.20) and 3.33 h (IQR 1.48–7.24) respectively. Initial HFNC flow was started at 60L/min (IQR 60–60). 41 patients (37%) had a second post-HFNC initiation ABG, only 22 of these had the ABG analysis more than 12 h after HFNC initiation.

**Table 1:** Baseline characteristics, comorbidities, and etiology of respiratory failure (n = 111)

	Total HFNC (n = 111)	HFNC success (n = 72)	HFNC failure (n = 39)	*P* value
**Patient demographics and characteristics**				
Age	66.0 (58.0–77.0)	64.0 (58.0–77.0)	69.0 (63.0–78.0)	0.009**
Male gender	85 (76.6%)	59 (81.9%)	26 (66.67%)	0.471
BMI	22.9 (19.2–27.3)	22.9 (19.0–27.3)	22.6 (19.5–27.6)	0.935
Surgical cases	21 (18.9%)	13 (18.1%)	8 (20.5%)	0.749
APACHE II	20.0 (15.0–27.0)	18.0 (12.0–23.0)	24.0 (20.0–30.0)	< 0.001**
SOFA Score	6.0 (4.0–9.0)	6.0 (4.0–8.8)	6.0 (4.0–9.0)	0.709
Vasopressor support at time of HFNC initiation	41 (36.9%)	26 (36.1%)	15 (38.5%)	0.810
**Comorbidities**				
Congestive Heart Failure	4 (3.6%)	4 (5.6%)	0 (0.0%)	0.134
Cancer	2 (1.8%)	0 (0.0%)	2 (5.1%)	0.052
Immunocompromised host	28 (25.2%)	20 (27.8%)	8 (20.5%)	0.401
COPD	2 (1.8%)	1 (1.4%)	1 (2.6%)	0.660
**Primary etiology for respiratory failure**				
Pneumonia	84 (75.7%)	53 (73.6%)	31 (79.5%)	0.490
Atelectasis	6 (5.4%)	5 (6.9%)	1 (2.6%)	0.332
Interstitial Lung Disease	3 (2.7%)	2 (2.8%)	1 (2.6%)	0.944
Septic Shock	9 (8.1%)	5 (6.9%)	4 (10.3%)	0.542
Others	9 (8.1%)	7 (9.7%)	2 (5.1%)	0.395

Values are expressed in number (percentage) and median (interquartile range)

^*^P value < 0.05; **P value < 0.01

*APACHE* Acute Physiology and Chronic Health Evaluation, *BMI* Body mass index, *COPD* Chronic obstructive pulmonary disease, *HFNC* High Flow Nasal Canula, *SOFA* Sequential Organ Failure Assessment

**Table 2:** Vitals, respiratory parameters, and outcomes (n = 111)

	Total HFNC (n = 111)	HFNC success (n = 72)	HFNC failure (n = 39)	*P* value
**Vital signs before the initiation of HFNC**				
GCS	15 (13–15)	15 (14–15)	15 (12–15)	0.223
Heart rate	103.0 (87.0–114.0)	103.0 (84.5–115.8)	105.0 (92.0–113.0)	0.834
Respiratory rate	26.0 (21.0–30.0)	25.0 (21.0–28.8)	27.0 (21.0–33.0)	0.238
SpO_2_, %	95.0 (91.0–97.0)	94.5 (91.0–97.0)	95.0 (91.0–97.0)	0.804
**Arterial blood gas analysis before the initiation of HFNC**				
pH	7.44 (7.38–7.47)	7.45 (7.40–7.47)	7.41 (7.37–7.47)	0.558
PaO_2_, mmHg	68.7 (59.0–80.0)	69.5 (58.7–81.8)	65.0 (59.0–74.5)	0.261
PF ratio	162.5 (112.0–228.0)	179.0 (126.5–268.3)	140.0 (100.0–176.0)	0.128
PaCO_2_, mmHg	32.6 (28.0–38.0)	32.0 (28.4–38.0)	33.0 (28.0–38.0)	0.640
SaO_2_, %	94.0 (92.0–97.0)	95.0 (92.0–97.0)	93.0 (91.0–96.0)	0.261
Bicarbonate, mmol/L	21.9 (18.7–25.4)	21.9 (18.9–25.2)	22.4 (18.7–26.0)	0.944
**HFNC settings and duration**				
Initial HFNC flow	60.0 (60.0–60.0)	60.0 (60.0–60.0)	60.0 (60.0–60.0)	1.000
Initial FIO_2_ set on HFNC	40.0 (33.0–80.0)	40.0 (35.0–50.0)	50.0 (40.0–60.0)	0.123
Duration of HFNC (hours)	18.2 (10.3–34.5)	26.1 (15.6–41.9)	9.6 (5.1–14.8)	< 0.001**
**Mortality outcome**				
Hospital Mortality	18 (16.2%)	7 (9.7%)	11 (28.2%)	< 0.001**
ICU Mortality	7 (6.3%)	1 (1.4%)	6 (15.4%)	0.004**

Values are expressed in number (percentage) and median (interquartile range)

^*^P value < 0.05; **P value < 0.01

*GCS* Glasgow coma score, *HFNC* High Flow Nasal Canula, *ICU* intensive care unit, *PF ratio* PaO_2_/FIO_2_ ratio

Of these 111 patients, 64.9% (72 patients) had HFNC success and 35.1% failed HFNC (39 patients; among them 32 were intubated and remaining 7 required NIV support). 82% of the failed HFNC patients had escalation of respiratory support within 24 h of HFNC initiation. HFNC failure was associated with a higher age and higher APACHE II score at ICU admission (Table 1). HFNC failure patients had poorer outcomes with higher ICU and hospital mortality.

Patients with HFNC failure had a significantly lower Pre- and Post-HFNC initiation POX, POX-HR and Delta POX-HR as well as ROX at all measured timepoints at 2, 6, 12, 18 h and post-HFNC initiation ROX as well as Delta ROX (Table 3).

**Table 3:** Variables and diagnostic accuracy for HFNC outcomes (n = 111)

		No. of patients who remain on HFNC	HFNC success	No. of patients who remain on HFNC	HFNC failure	*P* value	AUROC
ROX	Before Initiation of HFNC	72	8.83 (6.19–11.81)	39	6.81 (5.65–9.72)	0.075	0.604 (0.494–0.713)
	2 h	72	9.54 (7.77–12.97)	39	7.83 (6.33–10.43)	0.006**	0.659 (0.553–0.765)
	6 h	70	11.28 (9.58–14.86)	27	7.33 (6.11–9.80)	< 0.001**	0.759 (0.645–0.873)
	12 h	62	11.57 (9.36–13.61)	15	8.17 (6.53–10.56)	< 0.001**	0.767 (0.617–0.916)
	18 h	50	11.42 (9.67–13.89)	11	7.22 (5.39–10.33)	0.001**	0.815 (0.682–0.947)
	24 h	38	11.83 (8.65–15.61)	7	7.70 (5.54–12.93)	0.052	0.733 (0.525–0.941)
Post-HFNC initiation ROX^##^		72	10.16 (8.02–12.50)	39	8.04 (6.39–10.86)	0.036*	0.621 (0.505–0.737)
Delta ROX^##^		72	1.78 (-0.61–5.03)	39	0.32 (-3.65–1.85)	0.001**	0.690 (0.590–0.790)
POX	Pre-HFNC POX^#^	72	7.39 (5.04–11.33)	39	5.38 (3.60–7.00)	< 0.001**	0.705 (0.608–0.803)
	Post-HFNC POX^##^	72	8.47 (6.32–10.95)	39	5.96 (4.16–8.57)	< 0.001**	0.702 (0.594–0.811)
POX-HR	Pre-HFNC POX-HR^#^	72	8.87 (5.05–12.58)	39	5.28 (3.64–6.85)	< 0.001**	0.714 (0.619–0.810)
	Post-HFNC POX-HR^##^	72	9.35 (6.85–12.22)	39	5.98 (4.26–7.97)	< 0.001**	0.726 (0.622–0.829)
Delta POX-HR^##^		72	3.29 (0.61–5.34)	39	− 1.12 (− 3.99–1.63)	< 0.001**	0.813 (0.726–0.900)

^*^P value < 0.05; **P value < 0.01

^#^2.38 h (IQR 1.51–5.20) pre-HFNC initiation

^##^3.33 h (IQR 1.48–7.24) post-HFNC initiation

With regards to early prediction after HFNC initiation, Delta POX-HR at median 3.33 h after HFNC initiation (IQR 1.48–7.24) had the highest AUC of 0.813 (95% confidence interval (CI) 0.726–0.900) compared to various POX and POX-HR as well as ROX values at less than 12 h.

Delta POX-HR demonstrated a statistically significant AUROC value compared to ROX at 2 h (AUROC: 0.813 vs 0.659; p-value: 0.014), post-HFNC initiation ROX (AUROC: 0.813 versus 0.621; *p* value: 0.004) as well as Delta ROX (AUROC: 0.813 versus 0.690; p-value: 0.034). However, there was no significant difference between AUROC value of Delta POX-HR and post-HFNC POX, post-HFNC POX-HR as well ROX at 6, 12 and 18 h.

Using the AUROC curve, a cut-off of 0.1 was determined for Delta POX-HR for the prediction of HFNC success with the highest Youden Index (sensitivity 79.2%, specificity 71.8%). Similarly, the cut-off was 6.80 for post-HFNC POX-HR, with AUROC of more than 0.70 for the prediction of HFNC success (Table 4).

**Table 4:** Prediction of HFNC success based on Delta POX-HR cut offs

	Sensitivity (%)	Specificity (%)	NPV (%)	PPV (%)	LR +	LR−	Youden Index
Delta POX-HR > 0.10	79.2	71.8	65.1	83.8	2.81	0.29	0.510
Post-HFNC POX-HR > 6.80	77.8	64.1	59.5	79.7	2.17	0.46	0.409

*NPV* Negative predictive value, *PPV* Positive predictive value, *LR* Likelihood ratio

On univariate and multivariate COX proportional regression analysis, Delta POX-HR of > 0.10 and post-HFNC POX-HR > 6.80 were significantly associated with a lower risk of HFNC failure (Table 5). Other variables included in the multivariate analysis were age, gender, pre-HFNC POX-HR index, and APACHE II score.

**Table 5:** Cox regression analysis evaluating Delta POX-HR > 0.10 and post-HFNC POX-HR > 6.80 for the prediction of HFNC failure in patients with ARF

	Univariate Analysis	*P* value	Multivariate Analysis	*P* value
Delta POX-HR > 0.1	0.178 (0.088–0.360)	< 0.001**	0.236 (0.113–0.492)	< 0.001**
Post-HFNC POX-HR > 6.80	0.281 (0.084–0.748)	0.018*	0.363 (0.159–0.964)	0.042*

^*^P value < 0.05; **P value < 0.01

Other variables included in the multivariate analysis: age, gender, pre-HFNC POX-HR index, and APACHE II score

**Fig. 1:**
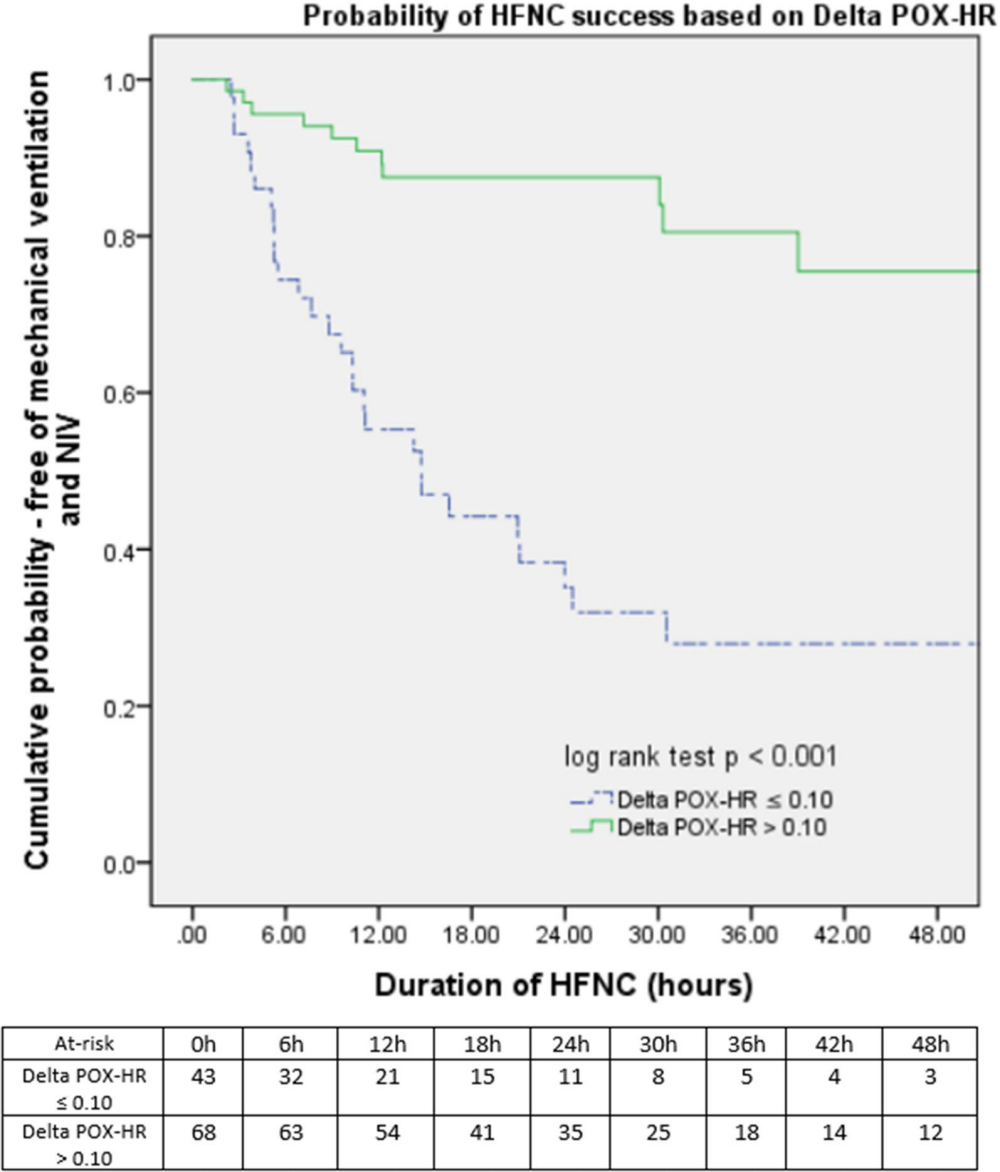
Kaplan–Meier plot for HFNC success probability based on Delta POX-HR for patients initiated on HFNC for acute respiratory failure

Kaplan–Meier estimates of the probability of HFNC success with a cut-off of 0.10 for Delta POX-HR index is shown in Fig. 1.

Conversely, we also investigated a cut off value for Delta POX-HR and post-HFNC POX-HR to predict higher risk of HFNC failure with > 90% specificity (Table 6).

**Table 6:** Cut-off for high risk of HFNC failure for change in POX-HR and post-HFNC POX-HR with > 90% specificity

	Cut-off	Sensitivity (%)	Specificity (%)	NPV (%)	PPV (%)	Youden Index
Delta POX-HR	− 1.20	46.2%	90.3%	75.6%	72.0%	0.365
Post-HFNC POX-HR	5.00	38.5%	91.7.0%	73.3%	71.4.0%	0.302

*NPV* Negative predictive value, *PPV* Positive predictive value

### Comparison of previous studies:

We evaluated the ROX index based on previously established cut-offs of 4.88 for HFNC success by Roca et al. [2, 3] at 2, 6 and 12 h (Table 7) and observed a low sensitivity at all the time-points. We then used specificity of at least 90% to find the ROX cut-off for our study population, and found that the cut-off for 2, 6 and 12 h were 5.92 (Sensitivity: 23.1%); 6.31 (Sensitivity: 33.3%); and 7.40 (Sensitivity: 40.0%) respectively.

**Table 7:** Comparison of Roca et al. cut-off for ROX using the ARF data in this study for HFNC success

	Sensitivity	Specificity	NPV	PPV
ROX at 2 h > 4.88	7.7%	100.0%	71.0%	100.0%
ROX at 6 h > 4.88	7.4%	98.6%	77.0%	100.0%
ROX at 12 h > 4.88	6.7%	100.0%	82.0%	100.0%

*NPV* Negative predictive value, *PPV* Positive predictive value

## Discussion

To our knowledge, ours is the first study demonstrating that a dynamic index incorporating heart rate and substitution of PF ratio for SF ratio in addition to respiratory rate (Delta POX-HR—change in pre- and post-HFNC initiation POX-HR) may facilitate early and accurate prediction of HFNC outcomes compared to previously validated ROX index among patients initiated on HFNC for ARF. Delta POX-HR was also statistically more significant compared to Delta ROX (calculated at same time as post-HFNC initiation blood gas being done).

Our results suggest that Delta POX-HR can predict HFNC outcomes very early, when the ability to predict HFNC failure and the need for escalation would be important. Previously, PF ratio within 6 h of HFNC initiation has been observed to be lower in HFNC failure group when compared to HFNC success group [7]; similarly, our proposed index can predict the outcomes early. Similar to our study, previous studies have demonstrated that most intubations occur between 10 to 24 h post-HFNC initiation [2, 3, 21]. Early intubation has been shown to be associated with better outcomes [5]. An early prediction can therefore avoid the delay in escalation of respiratory support and subsequent worse patient outcomes [5–7].

Our study results are similar to the recently published prospective observational study that demonstrated that incorporation of HR to modify ROX index may be a better predictor of HFNC failure compared to ROX alone [9]. Of note, as compared to the previous study, we planned to exclude the patients on beta-blocker and beta-2 agonist therapy. The rationale to exclude such patients was to avoid interference with HR response and subsequent POX-HR index performance. However, our study had only one such patient (less than 1% of study patients). Previous studies in general ICU population showed pre-existing beta blocker therapy usage in up to 4.6% of patients studied [22]. Small numbers in our study may be explained by the fact that it is common practice in our unit to withhold beta blockers in unwell patients. Similarly, usual practice in our unit is to start NIV rather than HFNC for patients with airway hyperreactivity who would have required beta-2 agonists. Additionally, apart from these common pharmacological agents, many other factors can interfere with HR response in critically unwell patients, like fever, baseline heart rate, anxiety. It may not be practically possible to differentiate between tachycardia due to cardiovascular compensation versus other pathophysiological stimuli like fever, anxiety. Therefore, we didn’t specifically exclude patients with such stimuli.

Moreover, the ROX index is a static measurement of clinical condition in ARF patients. Our dynamic index incorporating before and after-HFNC initiation parameters demonstrated better discrimination; even outperforming Delta ROX index. Interestingly, patients with HFNC failure in our study had significantly lower pre-HFNC initiation POX and POX-HR values. However, baseline respiratory rate, heart rate, FIO_2_, SpO_2_, PaO_2_, PF ratio as well as ROX index were similar. Our study was not designed to further analyse this finding. One may hypothesize that POX and POX-HR indices may be able to predict the outcomes even before starting HFNC, and the discrimination improves if the post-HFNC initiation parameters are incorporated. This finding needs further evaluation in future larger, multicentre prospective studies since we are unable to comment on clinicians’ rationale to use HFNC in such cases in our pragmatic retrospective study design.

PF ratio from ABG analysis is a commonly available physiological parameter among sick patients in respiratory failure in addition to HR and RR. Only 15 out of 237 screened patients (6.3%) in our study did not have ABGs done pre- or post-HFNC initiation. A recent large multi-center observational study of 1,161 patients initiated on HFNC usage noted that 85% patients had ABGs done before and after HFNC usage [19]. In view of clinical utility of our findings, we encourage clinicians to utilize PF ratio during HFNC usage in their decision making.

Our patient population and study results are similar to previous studies in many ways. Intubation outcome of 28.8% in the ARF patients is similar to previous studies (13–38.4%) [2, 3, 9]. ICU mortality was higher in HFNC failure group despite timely escalation of respiratory support within 12 h, consistent with previous studies.

Similar to previous studies, ROX index reliably predicted ARF patients at low risk of HFNC failure and the accuracy of ROX index was even better at 18 h among patients who were still on HFNC after 12 h [3, 9]. Of note, similar to previous studies, the cut off for ROX value that can predict HFNC success in our study was different to original ROX studies [2, 3, 8–11]. This could be explained by differences among studies in terms of study population (e.g., 75.7% pneumonia patients in our study compared to all patients having pneumonia of varying etiologies in the ROX development and validation studies [2, 3]), severity of illness (more patients with shock and a trend toward a higher APACHE II score in the multi-center validation study of ROX index [3]) and mixed intensive care unit (our study) compared to medical intensive care unit in other studies [9].

Inclusion of patients with ARF of varying etiologies in our study broadens the applicability of our results. Our study included escalation to any form of positive pressure respiratory support, intubation as well as NIV as an outcome measure rather than intubation alone, similar to another study [23]. Another strength of our study was that we used electronic medical records (EPIC®) to collect the data retrospectively. Therefore, we didn’t have any missing data and that adds to the robustness of our results.

However, several limitations exist. Firstly, selection of patients for HFNC and the determination of failed HFNC was not protocolized in the institution ICU and was based on clinical judgment of the ICU team. We used escalation to intubation or NIV as an objective parameter to address this issue of lack of standardization. In view of consultant led and round-the-clock respiratory therapist cover, we believe that any intubation/ NIV criteria would be applied equally to HFNC failure as well as success groups. Nonetheless, our intubation rate was comparable to previous studies. Similarly, recent reviews of HFNC use have identified similar limitations of lack of data and significant heterogeneity among the published studies to be able to guide clinical practice in this setting [24]. Secondly, ours is a single centre retrospective study comprising small number of patients. However, the ROX index was similarly developed in a small 157-patient study conducted at two centres [2] and a recent study that proposed modification of ROX score by incorporating the heart rate was a single centre study, comprising only 99 patients with ARF [9]. There is always a risk of overfitting in the studies with small sample size. We mitigated this risk by doing univariate analysis and selected variables that were both clinically and statistically significant for the multivariate analysis. However, this study would therefore need validation in larger studies. Additionally, there is risk of selection bias in retrospective studies, although we enrolled all consecutive patients started on HFNC for ARF during the study period. Of note, very small number of patients in our study were on beta blockers and beta-2 agonist therapy. Therefore, the modified index will not be applicable in patients receiving these agents, in view of interference with HR response.

## Conclusion

Our study results suggest dynamic Delta POX-HR index incorporating HR and substitution of PF ratio for SF ratio in addition to respiratory rate can facilitate early and accurate prediction of HFNC outcomes compared to ROX index as well as Delta ROX. Using the proposed cutoffs, both the Delta POX-HR > 0.10 and post-HFNC POX-HR > 6.80 perform well in predicting patients at low risk of HFNC failure among ARF patients, as early as 3 h into treatment. The findings of our retrospective study should be validated in larger prospective studies.

## Data Availability

The datasets used and/or analysed during the current study are available from the corresponding author on reasonable request.

## Abbreviations

ABG: Arterial blood gas
APACHE: Acute physiology and chronic health evaluation
ARF: Acute respiratory failure
BMI: Body mass index
GCS: Glasgow coma score
HR: Heart rate
ICU: Intensive care unit
IQR: Interquartile range
KM: Kaplan–Meier
NIV: Non-invasive ventilation
PF ratio: PaO_2_/FIO_2_
ROC curve: Receiver operating characteristic curve
ROX index: (SpO_2_/FIO_2_ ratio)/respiratory rate
SF ratio: SpO_2_/FIO_2_
SOFA: Sequential organ failure assessment
